# DNA repair gene polymorphisms and clinical outcome of patients with primary small cell carcinoma of the esophagus

**DOI:** 10.1007/s13277-014-2718-y

**Published:** 2014-11-06

**Authors:** Qiang Zhou, Bing-Wen Zou, Yong Xu, Jian-Xin Xue, Mao-Bin Meng, Fang-Jiu Liu, Lei Deng, Dai-Yuan Ma, Rui Ao, You Lu

**Affiliations:** 1 Department of Thoracic Oncology, Cancer Center and State Key Laboratory, West China Hospital, Medical School, Sichuan University, 37 Guoxue Lane, Chengdu, 610041 People’s Republic of China; 2 Department of Oncology, Suining Center Hospital, Suining, People’s Republic of China; 3 Department of Radiation Oncology, CyberKnife Center, Key Laboratory of Cancer Prevention and Therapy, Tianjin Medical University Cancer Institute and Hospital, National Clinical Research Center of Cancer, Tianjin, People’s Republic of China; 4 Division of Radiation and Cancer Biology, Department of Radiation Oncology, Stanford University School of Medicine, Stanford, CA USA; 5 Department of Radiotherapy Oncology, Affiliated Hospital of North Sichuan Medical College, Nanchong, People’s Republic of China; 6 Department of Oncology, Sichuan Province People’s Hospital, Chengdu, People’s Republic of China

**Keywords:** Clinical outcome, DNA repair gene, Esophageal small cell carcinoma, PARP1, XRCC1, XPC, BRCA1, BRCA2, Genetic polymorphism

## Abstract

Polymorphisms in DNA repair genes impact on the synthesis of DNA repair proteins that are crucial to the repair of DNA damages induced by chemotherapy and radiotherapy. We retrospectively examined whether there was an association between the selected six single nucleotide polymorphisms (SNPs) of five DNA repair genes (*PARP1-Val762Ala*, *XRCC1-Arg194Trp*, *XRCC1-Arg399Gln*, *XPC-Lys939Gln*, *BRCA1-Lys1183Arg*, and *BRCA2-Asn372His*) and the clinical outcome of patients with primary small cell carcinoma of esophagus (SCCE), and it showed that the median progression-free survival (PFS) and the overall survival (OS) were 11.8 versus 9.7 months (*P* = 0.041) and 17.4 versus 14.8 months (*P* = 0.032) for patients carrying the variant allele (T/C + C/C) and the wild-type allele (T/T) of *PARP1-Val762Ala* polymorphism, respectively. However, no statistical significance was observed in the other five polymorphic loci (*P* > 0.05). When these six SNPs were combined, however, patients with at least three variant genotypes had significantly longer PFS and OS compared with those carrying less than three variant genotypes (*P* = 0.009 and *P* = 0.007, respectively). The presence of at least three polymorphic variants in certain DNA repair genes may impact on patient survival and could be a potential genomic predictor of clinical response to DNA-damaging treatment in SCCE patients.

**Electronic supplementary material:**

The online version of this article (doi:10.1007/s13277-014-2718-y) contains supplementary material, which is available to authorized users.

## Introduction

Primary small cell carcinoma of esophagus (SCCE), which was first described by McKeown in 1952 [1], is the most common extrapulmonary small cell carcinoma in humans. It accounts for 0.05∼4 % of all esophageal malignancies [2]. The disease is characterized by a malignant biological behavior including aggressive progression, high incidence of metastasis, and poor prognosis [3]. On account of its rarity, the standard of therapeutic regimens for SCCE has not yet been established [4]. So far, however, the current therapeutic schedule for SCCE, similar to small cell lung cancer (SCLC), is a combination treatment, including surgery, chemotherapy, and radiotherapy, as reported for SCCE by Song et al. [5]. One major effect of both chemotherapy and radiotherapy is the induction of DNA damages, including formation of single-strand breaks (SSBs) and double-strand breaks (DSBs).

Considering that both DNA-damaging agents such as platinum and radiation can be used for the treatment of cancer, we hypothesized that the DNA repair capacity of cancer cells influenced the clinical outcome of diverse types of cancer, including SCCE. Actually, one of the most important mechanisms of resistance to cytotoxic anticancer drugs and radiation, as we have known it, is the highly efficient DNA repair capacity of cancer cells, which increases tolerance to DNA damages induced by chemotherapy [6] and radiotherapy [7]. Previous studies have also shown that genetic variations in DNA repair genes may influence DNA repair capacity. As the most common type of genetic variation, single nucleotide polymorphisms (SNPs) in DNA repair genes can influence the synthesis of DNA repair proteins, alter the functional properties of DNA repair proteins [8], and result in DNA repair deficiency, which is associated with chemotherapy and radiotherapy sensitization [9]. Different clinical outcomes of several types of cancers are partially due to interindividual variations of DNA repair activity. In other words, SNPs of DNA repair genes may influence the therapeutic efficacy of chemotherapy and radiotherapy. Up to the present, many DNA repair gene polymorphisms have been shown to be associated with cancer prognosis [10] despite the fact that their biologic significance has not yet been fully elucidated. To explore the relationship between DNA repair gene polymorphisms and the clinical outcome of SCCE patients, we investigated the polymorphisms of DNA repair genes poly(ADP-ribose) polymerase 1 (PARP1) and X-ray repair cross-complementing protein 1 (XRCC1), which are involved in base excision repair (BER), breast cancer susceptibility gene 1 (BRCA1) and breast cancer susceptibility gene 2 (BRCA2), which are involved in homologous recombination repair (HR), and xeroderma pigmentosum group C (XPC), which is involved in nucleotide excision repair (NER).

To our knowledge, few studies have addressed DNA repair gene polymorphisms and their relationship with the clinical outcome of malignant tumors of the esophagus, especially SCCE. Accordingly, in the study, we retrospectively analyzed the status of the PARP1-Val762Ala, XRCC1-Arg194Trp, XRCC1-Arg399Gln, XPC-Lys939Gln, BRCA1-Lys1183Arg, and BRCA2-Asn372His gene polymorphisms and their possible impact alone or in combination on progression-free survival (PFS) and overall survival (OS) in patients with SCCE.

## Patients and methods

### Study subjects

We retrospectively reviewed the clinicopathologic data of patients with pathologically confirmed SCCE who received surgical treatment at four cancer centers in China (Appendix) between January 2000 and August 2010. SCCE was diagnosed by histological examinations according to immunohistochemical criteria [2]. The inclusion criteria were as follows: (1) having been diagnosed with an immunopathologically confirmed SCCE after esophagectomy and (2) having available tissue samples for DNA extraction. The main exclusion criteria were the presence of preoperative chemotherapy or radiotherapy because of their potential influence on results.

The study protocol and acquisition of tissue specimens were approved by the local institutional review boards at the authors’ affiliated institutions and carried out with the state regulations regarding the experimental use of human tissues. Patient consent was not required because of the retrospective nature of the study.

### Treatments

Chemotherapeutic regimens were as follows: (1) cisplatin 40 mg/m^2^ on days 1–3 and etoposide 60 mg/m^2^ on days 1–3, every 3 weeks; (2) carboplatin AUC 5 or 6 on day 1 and etoposide 60 mg/m^2^ on day 1, every 3 weeks; (3) cisplatin 40 mg/m^2^ on days 1–3, leucovorin 200 mg/m^2^ on days 1–5, and fluorouracil 750 mg/m^2^ on days 1–5, every 3 weeks; (4) cisplatin 40 mg/m^2^ on days 1–3 and paclitaxel 135 mg/m^2^ on day 1, every 3 weeks; (5) cisplatin 40 mg/m^2^ on days 1–3 and vinorelbine 25 mg/m^2^ on days 1 and 8, every 3 weeks; (6) cisplatin 40 mg/m^2^ on days 1–3, cyclophosphamide 600 mg/m^2^ on day 1, and doxorubicin 40 mg/m^2^ on day 1, every 3 weeks; (7) oxaliplatin 85 mg/m^2^ on day 1 and docetaxel 75 mg/m^2^ on day 1, every 3 weeks.

Radiotherapy programs include conventional radiotherapy, three-dimensional conformal radiotherapy and intensity-modulated radiation therapy with six to eight MV-X rays. The radiation dose ranged from 44 to 52 Gy, 1.8–2 Gy per fraction, 5 days per week, with a median dose of 50 Gy.

### Selection of candidate SNPs

Six SNPs in DNA repair genes [11] were selected according to the following criteria: (1) the minor allele frequency (MAF) is greater than 5 % [12] among Chinese, (2) SNPs belong to missense mutation which could affect protein function, and (3) SNPs have been reported previously to be associated with cancer risk or clinical outcome.

### Genotyping

Genomic DNA was extracted from dissected formalin-fixed paraffin-embedded (FFPE) tumor tissue blocks using the QIAamp DNA FFPE Tissue Kit (Qiagen, Hilden, Germany) according to the manufacturer’s instruction. DNA sequences including each SNP of interest were amplified from the genomic DNA by polymerase chain reaction (PCR) as reported before [13] using commercial PCR Amplification Kit (TaKaRa, Dalian, China). After amplification, the purified PCR fragments were directly sequenced using the dideoxy chain termination method of sequencing.

### Statistical analysis

Clinical and follow-up data were retrospectively obtained through inpatient and outpatient records or by contacting patients and their families. Follow-up began at diagnosis, and patients were censored at the time of death, loss to follow-up, or the end of follow-up. PFS was calculated from the date of diagnosis to the date of disease progression or to the date of death of any cause. PFS was also censored at the time of the last follow-up when any patient had not progressed or was still alive. OS was calculated from the date of diagnosis to the date of death of any cause or to the date of the last follow-up.

The association between PFS or OS and the PARP1-Val762Ala, XRCC1-Arg194Trp, XRCC1-Arg399Gln, XPC-Lys939Gln, BRCA1-Lys1183Arg, and BRCA2-Asn372His genotypes was estimated using Kaplan-Meier curves and log-rank test. For the analysis of the combination effect of the five DNA repair gene polymorphisms on PFS and OS, the total number of variant genotypes from them was also estimated using the Kaplan-Meier method. Cox proportional hazards models were used to analyze the association of DNA repair gene polymorphisms and clinical factors for PFS and OS. Data were analyzed using the SPSS software version 17.0 (SPSS Inc., Chicago, IL). All statistical tests were two sided, and the level of 0.05 was considered statistically significant.

## Results

### Patient demographic and baseline characteristics

**Fig. 1:**
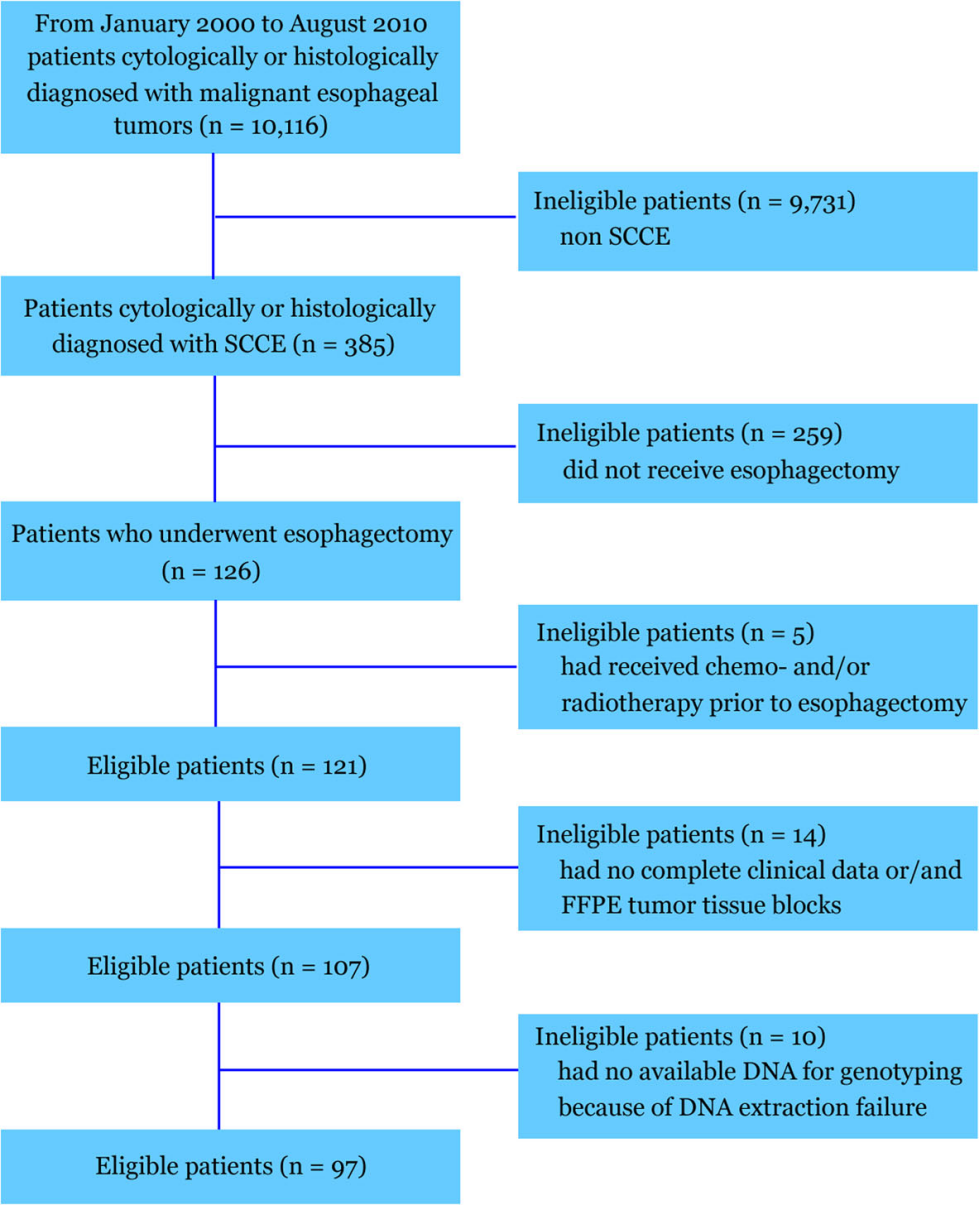
CONSORT diagram. *SCCE* small cell carcinoma of esophagus

Patient demographic and baseline characteristics are summarized in Table 1. More than 10,000 patients with pathologically confirmed malignant tumors of the esophagus were recruited. Among these patients, we identified a subset of 121 patients with SCCE, which was confirmed using the immunohistochemical criteria for the diagnosis of SCCE. All these 121 patients had received no chemotherapy and/or radiotherapy prior to esophagectomy. One hundred and seven patients had both FFPE tumor tissue blocks and complete clinical data including age, gender, smoking history, alcohol history, tumor location, stage, Eastern Cooperative Oncology Group (ECOG) performance status, and treatment. Ten patients had no available DNA for genotyping because of DNA extraction failure. Finally, 97 patients were included in the study. The study flow chart is shown in Fig. 1. Their median age was 59 years (range, 36 to 75). The majority (81.4 %) of the patients were men. Ten (10.3 %) patients had stage I, 19 (19.6 %) had stage IIA, 18 (18.6 %) had stage IIB, 38 (39.2 %) had stage III, and 12 (12.4 %) had stage IV SCCE. The follow-up ended on March 31, 2011, with a median follow-up of 65 months for those who were still alive (range, 9.1–116 months).

**Table 1:** Patient demographic and baseline characteristics

Parameter	No. of patients (%)
Age (years)	
≥60	50 (51.5)
<60	47 (48.5)
Gender	
Male	79 (81.4)
Female	18 (18.6)
ECOG performance status	
0	39 (40.2)
1	35 (36.1)
2	23 (23.7)
Tumor location	
Ut	5 (5.2)
Mt	57 (58.7)
Lt	35 (36.1)
Smoking history	
Nonsmoker	25 (25.8)
Smoker	72 (74.2)
Alcohol history	
Never	9 (9.3)
Previous (≥6 months)	33 (34.0)
Current	55 (56.7)
Postoperative stage	
I	10 (10.3)
IIa	19 (19.6)
IIb	18 (18.6)
III	38 (39.2)
IV	12 (12.4)
Treatment	
S + C	31 (32.0)
S + R	16 (16.5)
S + CR	50 (51.5)
Chemotherapy regimens	
EP	47/81 (58.0)
No-EP regimens	
PFL	10/81 (12.3)
PT	11/81 (13.6)
VP	4/81 (4.9)
CAP	7/81 (8.6)
Oxaliplatin + docetaxel	2/81 (2.3)
Radiotherapy regimens	
Conventional radiotherapy	19/66 (28.8 %)
3-DCRT	26/66 (39.4 %)
IMRT	21/66 (31.8 %)
Radiation dose (Gy)	
≥50	52/66 (78.8 %)
<50	14/66 (21.2 %)

*ECOG* Eastern Cooperative Oncology Group, *Ut* upper thoracic esophagus, *Mt* middle thoracic esophagus, *Lt* lower thoracic esophagus, *S* surgery, *C* chemotherapy, *R* radiotherapy, *CR* chemoradiotherapy, *EP* cisplatin/carboplatin + etoposide, *PFL* cisplatin + leucovorin + fluorouracil, *PT* cisplatin + paclitaxel, *VP* cisplatin + vinorelbine, *CAP* cisplatin + cyclophosphamide + doxorubicin, *3-DCRT* three-dimensional conformal radiotherapy, *IMRT* intensity-modulated radiation therapy

All the eligible patients underwent esophagectomy. Thirty-one (32.0 %) and 16 (16.5 %) patients received adjuvant chemotherapy and radiotherapy, respectively. Fifty (51.5 %) patients received adjuvant chemoradiotherapy. Among those patients who received radiotherapy alone or in combination with chemotherapy, 19 (28.8 %) patients received conventional radiotherapy, 26 (39.4 %) patients received three-dimensional conformal radiotherapy, and the remaining 21 (31.8 %) patients received intensity-modulated radiation therapy.

Our univariate analysis showed that age, sex, performance status on the Eastern Cooperative Oncology Group scale, tumor location, smoking history, and alcohol history were not significantly associated with PFS and OS (Supplemental Table S1). At the end of our study (March 31, 2011), 81 (83.5 %) patients died. The median follow-up duration for those who were still alive at the final follow-up was 65 months (range, 9.1 to 116). The median PFS was 10.2 months (95 % confidence interval (CI), 7.6 to 12.7), and the median OS was 15.9 months (95 % CI, 12.3 to 19.4). The 1-, 3-, and 5-year survival rates were 59.8, 21.6, and 13.4 %, respectively.

### Genotype frequencies

Genotype frequencies of 97 SCCE patients are presented in Table 2. The variant allele frequency for PARP1-Val762Ala, XRCC1-Arg194Trp, XRCC1-Arg399Gln, XPC-Lys939Gln, BRCA1-Lys1183Arg, and BRCA2-Asn372His polymorphisms was 34, 24.2, 25.7, 31.4, 37.1, and 13.9 %, respectively. In addition, 2 (2 %), 11 (11.3 %), 26 (26.8 %), 28 (28.8 %), 21 (21.6 %), and 9 (9.3 %) patients harbored from zero to five variant genotypes at any of the SNPs tested, respectively. All these genotype frequencies were found to be in Hardy-Weinberg equilibrium. No association was detected between the six SNPs and age, sex, ECOG performance status, tumor location, smoking history, alcohol history, and treatment regimen (Supplemental Tables S2-1 to S2-6).

**Table 2:** Association of genotypes with PFS and OS

Genotype	No. of patients (%)	MAF	PFS Median (95 % CI)	Log-rank *P*	OS Median (95 % CI)	Log-rank *P*
PARP1-Val762Ala		0.34				
T/T	43 (44.3)		9.7 (6.7–12.6)	0.121	14.8 (8.6–20.9)	0.098
T/C	42 (43.3)		10.1 (4.4–15.7)		15.4 (8.7–22.0)	
C/C	12 (12.4)		11.8 (8.5–15.0)		18.8 (12.3–25.2)	
T/C + C/C	54 (55.7)		11.8 (8.3–15.2)	0.041	17.4 (12.0–22.7)	0.032
T/T	43 (44.3)		9.7 (6.7–12.6)		14.8 (8.6–20.9)	
XRCC1-Arg194Trp		0.242				
C/C	54 (55.7)		10.2 (6.9–13.4)	0.788	15.9 (9.3–22.4)	0.521
C/T	39 (40.2)		10.0 (6.0–13.9)		15.4 (10.9–19.8)	
T/T	4 (4.1)		12.5 (0.0–26.4)		14.8 (−)	
C/T + T/T	43 (44.3)		10.1 (6.1–14.0)	0.999	15.4 (11.2–19.5)	0.829
C/C	54 (55.7)		10.2 (6.9–13.4)		15.9 (9.3–22.4)	
XRCC1-Arg399Gln		0.257				
G/G	52 (53.6)		9.7 (5.5–13.8)	0.753	14.8 (10.6–18.9)	0.687
G/A	40 (41.2)		11.9 (7.8–15.9)		18.3 (13.4–23.1)	
A/A	5 (5.2)		13.9 (0.0–30.8)		22.1 (0.0–53.4)	
G/A + A/A	45 (46.4)		12.5 (8.5–16.4)	0537	18.7 (14.2–23.1)	0.485
G/G	52 (53.6)		9.7 (5.5–13.8)		14.8 (10.6–18.9)	
XPC-Lys939Gln		0.314				
A/A	49 (49.5)		9.0 (6.6–11.3)	0.296	13.5 (8.0–18.9)	0.268
A/C	35 (37.1)		11.8 (8.5–15.0)		18.0 (13.3–22.6)	
C/C	13 (13.4)		23.4 (7.0–39.7)		33.4 (4.3–62.4)	
A/C + C/C	48 (50.5)		11.9 (7.0–16.7)	0.168	18.8 (14.4–23.1)	0.185
A/A	49 (49.5)		9.0 (6.6–11.3)		13.5 (8.0–18.9)	
BRCA1-Lys1183Arg		0.371				
A/A	38 (39.2)		8.6 (5.8–11.3)	0.578	13.5 (8.5–18.4)	0.324
A/G	46 (47.4)		11.8 (5.0–18.5)		18.8 (15.1–22.4)	
G/G	13 (13.4)		12.8 (2.4–23.1)		19.9 (2.7–37.0)	
A/G + G/G	59 (60.8)		12.5 (7.6–17.3)	0.303	19.2 (16.9–21.4)	0.15
A/A	38 (39.2)		8.6 (5.8–11.3)		13.5 (8.5–18.4)	
BRCA2-Asn372His		0.139				
T/T	70 (72.2)		9.7 (6.7–12.6)	0.147	14.9 (9.3–20.4)	0.154
T/G	27 (27.8)		11.9 (8.6–15.1)		18.8 (12.8–24.7)	

*MAF* minor allele frequency, *PFS* progression-free survival, *OS* overall survival, *CI* confidence interval

### Association of genotypes with PFS and OS

**Fig. 2:**
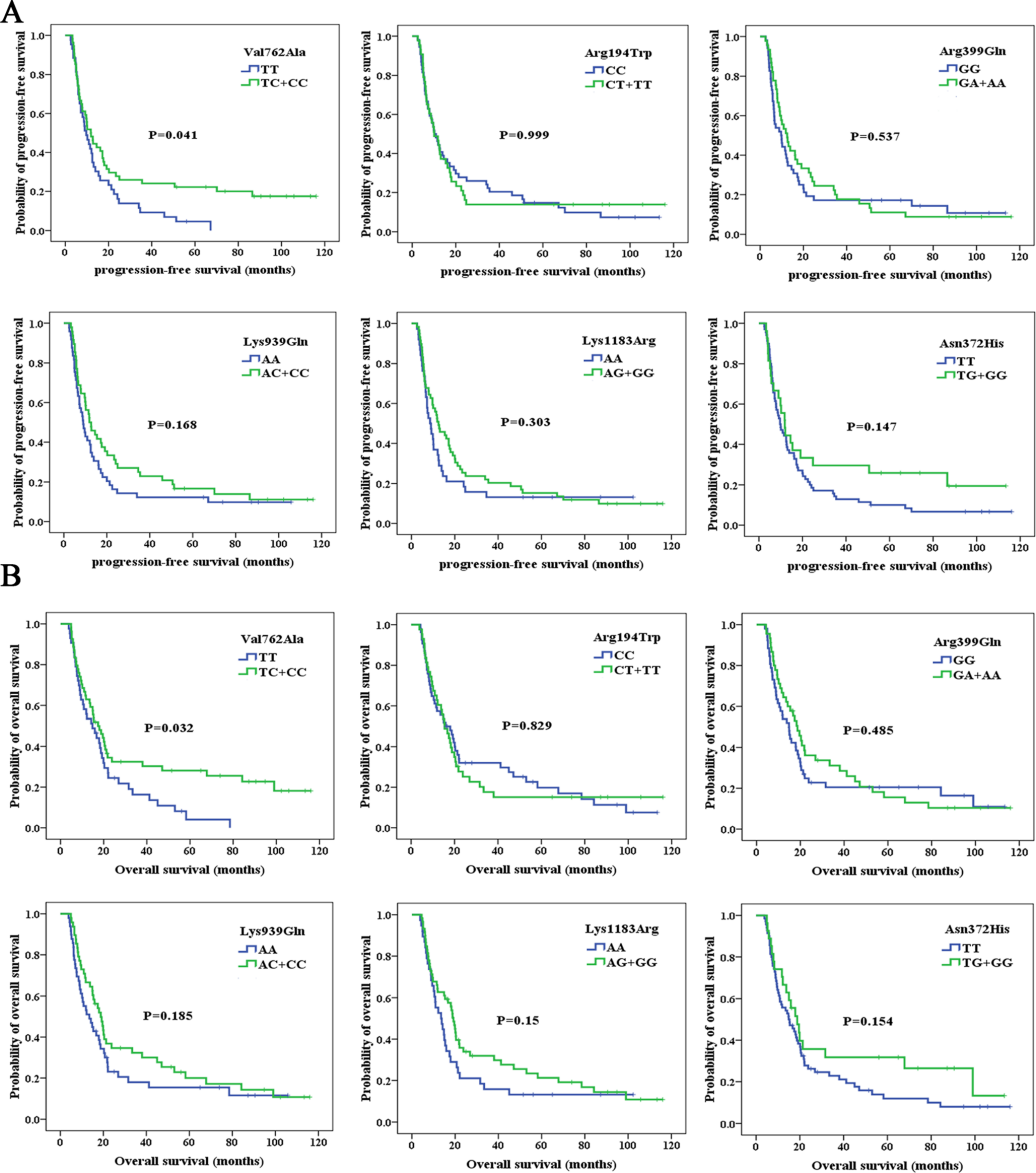
Progression-free survival (PFS) (**a**) and overall survival (OS) (**b**) stratified by genotypes (PARP1-Val762Ala, XRCC1-Arg194Trp, XRCC1-Arg399Gln, XPC-Lys939Gln, BRCA1-Lys1183Arg, and BRCA2-Asn372His)

To test the effect on the prognosis of DNA repair gene SNPs, we compared the PFS and OS of the patients carrying wild-type genotypes with those of patients carrying variant genotypes. For PARP1-Val762Ala, the frequencies of homozygous wild-type genotype (T/T), heterozygous (T/C), and homozygous mutant genotype (C/C) were 44.3, 43.3, and 12.4 %, respectively (Table 2). The median PFS for those patients carrying the variant allele (T/C + C/C) was 11.8 months (95 % CI, 8.3 to 15.2), and the median PFS for those patients carrying the wild-type allele (T/T) was 9.7 months (95 % CI, 6.7 to 12.6) (Table 2, Fig. 2a). The difference was statistically significant (*P* = 0.041). Similarly, the median OS for those patients carrying the variant allele (T/C + C/C) was 17.4 months (95 % CI, 12.0 to 22.7), and the median OS for those patients carrying the wild-type allele (T/T) was 14.8 months (95 % CI, 8.6 to 20.9) (Table 2, Fig. 2b). The difference was statistically significant (*P* = 0.032). However, no statistically significant association was observed between each of the other five selected SNPs and PFS and OS (Table 2, Fig. 2).

### Combined effects of six SNPs on PFS and OS

**Fig. 3:**
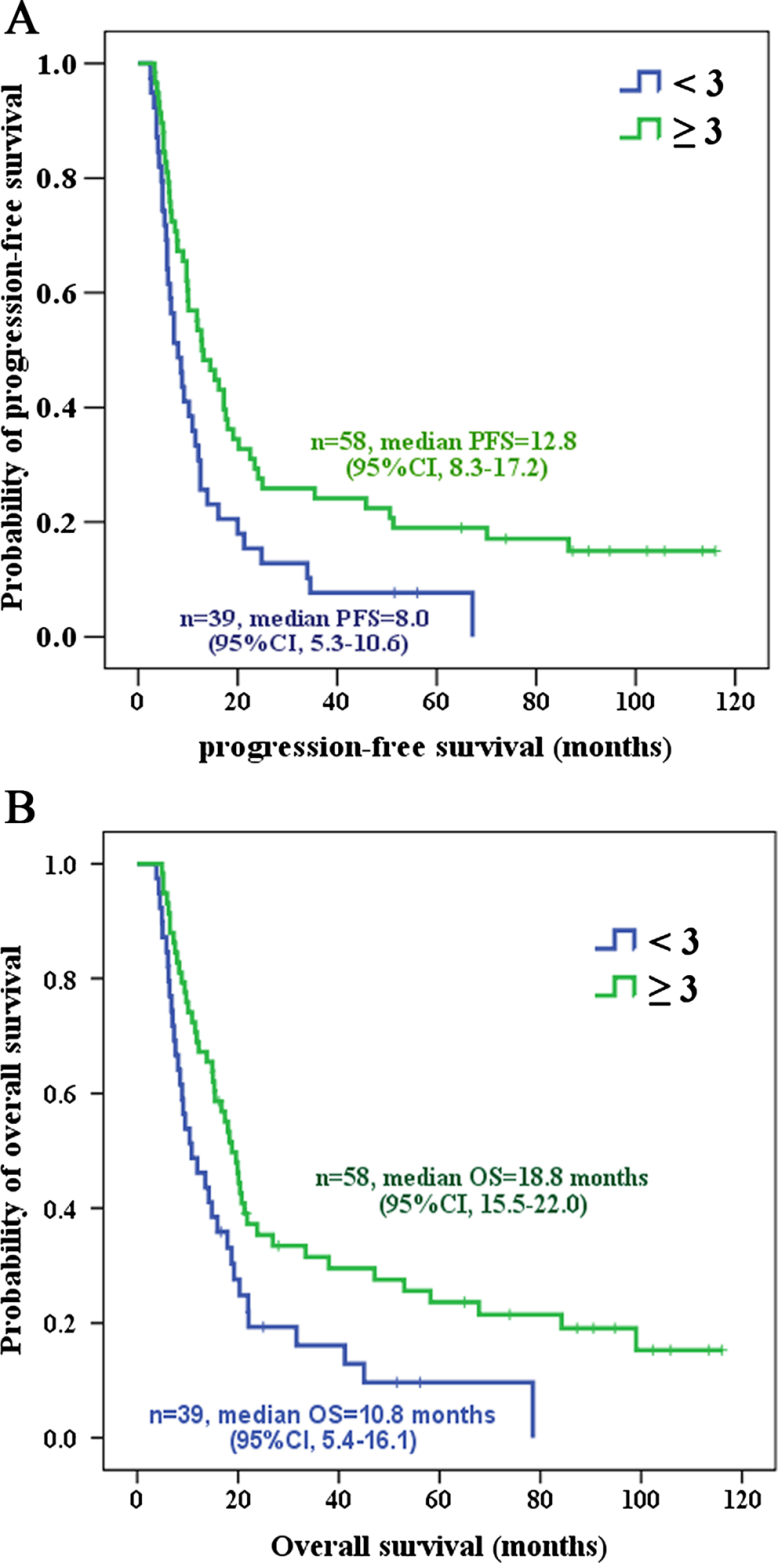
Combined effects of six genotypes on PFS (**a**) and OS (**b**). The Kaplan-Meier method was used to assess the combined effects of the PARP1-Val762Ala, XRCC1-Arg194Trp, XRCC1-Arg399Gln, XPC-Lys939Gln, BRCA1-Lys1183Arg, and BRCA2-Asn372His genotypes on PFS and OS. *P* = 0.009 for PFS and *P* = 0.07 for OS, log-rank test

Although no statistically significant association was observed between each of the five SNPs (except PARP1-Val762Ala) and PFS and OS, a statistically significant difference was observed when the six SNPs were analyzed in combination. Those patients with at least three variant genotypes had significantly longer PFS and OS compared with those who had less than three variant genotypes (12.8 vs. 8.0 months, *P* = 0.009 and 18.8 vs. 10.8 months, *P* = 0.007, respectively) (Tables 3 and 4, Fig. 3a, b). In the Cox proportional hazards model, we found that the risk of tumor progression was significantly lower for those patients carrying at least three variant genotypes (hazard ratio (HR) = 0.45; 95 % CI, 0.29 to 0.7; *P* = 0.0001) compared with those carrying less than three variant genotypes (Table 3). Similarly, those patients with at least three variant genotypes had markedly lower death risk (HR = 0.43; 95 % CI, 0.27 to 0.68; *P* = 0.0001) compared with those carrying less than three variant genotypes (Table 4).

**Table 3:** Combined effects of the variant genotypes on PFS

No. of variant genotypes	No. of patients (%)	PFS Median (95 % CI)	Univariate analysis		Multivariate analysis	
			LR	*P*	HR (95 % CI)	*P*
<3	39 (40.2)	8.0 (5.3–10.6)	6.9	0.009	1 (reference)	0.0001
≥3	58 (59.8)	12.8 (8.3–17.2)			0.45 (0.29–0.7)	

*PFS* progression-free survival, *CI* confidence interval, *LR* log-rank, *HR* hazard ratio

**Table 4:** Combined effects of the variant genotypes on OS

No. of variant genotypes	No. of patients (%)	OS Median (95 % CI)	Univariate analysis		Multivariate analysis	
			LR	*P*	HR (95 % CI)	*P*
<3	39 (40.2)	10.8 (5.4–16.1)	7.2	0.007	1 (reference)	0.0001
≥3	58 (59.8)	18.8 (15.5–22.0)			0.43 (0.27–0.68)	

*OS* overall survival, *CI* confidence interval, *LR* log-rank, *HR* hazard ratio

## Discussion

Efficient DNA repair plays a crucial role in maintaining genomic integrity and stability in mammalian cells [14]. There are at least six pathways whereby damaged DNA can be repaired [15], such as BER, NER, and HR. Loss of these DNA repair pathways can lead to genetic instability and predisposition to cancer. For instance, women with defective HR genes BRCA1 and BRCA2 have a significantly increased risk of developing breast cancer or ovarian cancer [16]. Animal experiments have also shown that mice deficient in BER were predisposed to lung cancer [17]. However, DNA repair may be regarded as a double-edged sword [18] because decreased DNA repair may not only play a pivotal role in carcinogenesis but also simultaneously increase sensitivity to DNA-damaging agents and consequently improve survival in cancer patients treated with DNA-damaging agents [19–21]. Thus, DNA repair genes may be used clinically as potential predictors for response to DNA-damaging treatment such as chemotherapy and radiotherapy or as prognostic factors for survival.

SNPs are the most common type of germline genetic variations, and previous research indicates that SNPs in DNA repair genes may influence the synthesis of DNA repair protein and decrease DNA repair protein activities [8] and may be important predictors for DNA repair capacity [22]. Furthermore, Wang et al. found that all homozygous variant genotypes of XP (except for XPG) had the lowest DNA repair capacity [22]. Based on these, in the current study, we aimed to identify whether there is an association between the selected DNA repair SNPs and the clinical outcome of SCCE patients.

The PARP1 gene encodes poly(ADP-ribose) polymerase 1 (PARP1), a key BER protein activated by DNA damage and involved in SSB repair [23]. The commonest polymorphism for the PARP1 gene is a single T to C substitution at nucleotide 762 (T to C; rs1136410), which leads to the replacement of valine by alanine, resulting in a decreased enzymatic activity of PARP1 [8]. The XRCC1 gene is another important member of the BER pathway, and the most extensively investigated coding region SNPs include Arg194Trp on exon 6 (C to T; rs1799782) and Arg399Gln on exon 10 (G to A; rs25487) [24]. The XPC gene participates in NER, and Lys939Gln on exon 15 (A to C; rs2228001) is the extensively investigated coding region SNP in the XPC gene [25]. In vitro study indicated that the C allele of the XPC Lys939Gln was associated with depressed levels of NER [26]. Both BRCA1 and BRCA2 play important roles in HR, which is involved in the repair of DSBs, and carriers of mutant BRCA1 or BRCA2 genes have favorable responses to carboplatin or cisplatin [19, 21].

Studies on the association of DNA repair gene polymorphisms with clinical outcome of cancer patients have yielded conflicting results. In an analysis of 103 patients with stage IIIA–IV lung cancer, those with one or two variant alleles at XRCC1 (Arg399Gln) or XPD (Asp312Asn) showed poorer OS compared with those carrying homozygous wild-type alleles (*P* = 0.07 and *P* = 0.003, respectively). Furthermore, a combined analysis of these two SNPs showed that more variant genotypes were associated with decreasing OS (*P* = 0.009) [27]. Conversely, Quintela-Fandino et al. concluded that the accumulation of polymorphic variants including XRCC1 (Arg399Gln) predicts a favorable clinical outcome among patients with advanced head and neck squamous cell carcinoma (HR = 2.94, *P* = 0.041) [28]. Our study, however, indicates that there was no significant association between each of the SNPs examined (except PARP1-Val762Ala) and PFS or OS of patients with primary SCCE (Table 2, Fig. 1). When we analyzed the combined effects of the six SNPs on PFS and OS, however, we found a statistically significant association between the number of variant genotypes (≥3) and PFS or OS (*P* = 0.009 and *P* = 0.007, respectively; Tables 3 and 4, Fig. 3a, b). Those patients with at least three variant genotypes had markedly longer PFS and OS compared with those who had less than three variant genotypes (PFS, 12.8 vs. 8.0 months; OS, 18.8 vs. 10.8 months).

To our knowledge, we report for the first time the role of six DNA repair SNPs in the clinical outcome of SCCE patients, and our results indicate that the five SNPs showed no significant effect individually except PARP1-Val762Ala, but the combined genotype effect of these six SNPs on PFS and OS was dramatic. These results may be easy to understand because in the complex networks of DNA repair, each mutation in a gene can affect only a part of DNA repair functions, and one repair pathway can complement for the other, such as BER and HR [29]. Thus, our findings suggest that these SNPs may act in concert, and the combined action of these SNPs may have a greater influence on the phenotype than each individual SNP, strongly indicating the importance of multiple SNPs in DNA repair genes as a determinant for DNA-damaging response to genotoxic therapy.

Although resistance to cancer chemotherapy and radiotherapy may be regulated by multiple factors and the mechanism of resistance is very complex, as previous investigations have shown [7, 30, 31], enhanced DNA repair may be one of the most important resistance mechanisms. The results we present herein suggest that patients carrying more variant genotypes of DNA repair genes have a favorable clinical outcome; in other words, patients with homozygous wild-type alleles have a poorer outcome probably due to their efficient DNA repair capability and resultant resistance to chemotherapy and radiotherapy. Individuals with cancer who have sequence variant in DNA repair genes may respond differently to DNA-damaging treatment. Therefore, combined DNA repair polymorphisms rather than single SNP are likely to be one of the most important determinants of individual responses to DNA-damaging treatment. Identification of DNA repair gene polymorphisms may be important to develop individualized treatment, and would be of potential value in predicting therapeutic outcomes in clinical practice [32].

There are some limitations in this study. Firstly, this is a retrospective study. The effects of disease stage and different therapeutic regimens on survival may affect our results, and retrospective recruitment of patients might lead to survival bias. Secondly, we only investigated six candidate gene polymorphisms that are thought to be functional based on previous studies [11], while other DNA repair genes were not considered, and therefore, we cannot evaluate the entirety of polymorphic variation across the total landscape of DNA repair genes. It also requires further investigation whether other polymorphisms in DNA repair genes affect the responses to DNA-damaging treatment in different tumor types. Thirdly, our sample size is small, and the statistical power of the study was limited. Fourthly, we tested this hypothesis only among a Chinese population, and our findings may not be applicable to other ethnic groups. Finally, our study lacked the measurement of expression levels of these five DNA repair genes and that we could not exactly determine the association between genotypes and phenotypes of these candidate genes. Based on the above analysis, our findings should be interpreted carefully.

In conclusion, we have demonstrated an association between multiple DNA repair gene polymorphisms and clinical outcome of SCCE patients. These SNPs of DNA repair genes may serve as predictive markers for responses to cancer DNA-damaging treatment. Further prospective and multi-institutional studies are warranted to confirm these conclusions in SCCE and other cancers.

### Electronic supplementary material

Below is the link to the electronic supplementary material.

Table S1 (DOCX 33 kb)

Table S2-1 (DOCX 30 kb)

Table S2-2 (DOCX 30 kb)

Table S2-3 (DOCX 30 kb)

Table S2-4 (DOCX 30 kb)

Table S2-5 (DOCX 30 kb)

Table S2-6 (DOCX 30 kb)

## Appendix I

1. Department of Thoracic Oncology, Cancer Center, West China Hospital, Medical School, Sichuan University, Chengdu, People’s Republic of China
2. Department of Oncology, Suining Center Hospital, Suining, People’s Republic of China
3. Department of Radiotherapy Oncology, Affiliated Hospital of North Sichuan Medical College, Nanchong, People’s Republic of China
4. Department of Oncology, Sichuan Province People’s Hospital, Chengdu, People’s Republic of China

## Abbreviations

SCCE: Small cell carcinoma of esophagus
SNPs: Single nucleotide polymorphisms
PARP1: Poly(ADP-ribose) polymerase 1
XRCC1: X-ray repair cross-complementing protein 1
XPC: Xeroderma pigmentosum group C
BRCA1/2: Breast cancer susceptibility gene 1/2
PFS: Progression-free survival
OS: Overall survival
