# Supplementary material for: DNA repair gene polymorphisms and clinical outcome of patients with primary small cell carcinoma of the esophagus
Source: Tumour Biol. 2014 Nov 6;36(3):1539–48. doi: 10.1007/s13277-014-2718-y (PMC4375303; doi:10.1007/s13277-014-2718-y)
Supplement: Supplementary file 1 — (DOCX 33 kb) [file 13277_2014_2718_MOESM1_ESM.docx]

Supplemental Table S1 Association of [clinicopathologic characteristics](http://www.google.com.hk/url?sa=t&rct=j&q=clinical+Pathology+Characteristics&source=web&cd=2&cad=rja&ved=0CEEQFjAB&url=%68%74%74%70%3a%2f%2f%77%77%77%2e%6e%63%62%69%2e%6e%6c%6d%2e%6e%69%68%2e%67%6f%76%2f%70%75%62%6d%65%64%2f%32%33%32%39%32%31%31%39&ei=BaxdUb6yJ46FiAeprYCwCA&usg=AFQjCNELnXrCiE-QnqGDzbreLj8VLaZNrA&bvm=bv.44770516,d.aGc) with PFS and OS

| Parameter | Median PFS, mo (95% CI) | *P* | Median OS, mo (95% CI) | *P* |
| --- | --- | --- | --- | --- |
| Age, years |  | 0.388 |  | 0.395 |
| ≥ 60 | 9.0(6.6-11.3) |  | 13.5(9.4-17.5) |  |
| < 60 | 12.1(10.3-13.8) |  | 19.2(15.6-22.7) |  |
| Gender |  | 0.896 |  | 0.985 |
| Male | 10.1(7.2-12.9) |  | 15.4(10.6-20.1) |  |
| Female | 10.2(4.7-15.6) |  | 15.9(9.6-22.1) |  |
| ECOG PS |  | 0.871 |  | 0.801 |
| 0 | 11.5(7.2-15.7) |  | 17.4(10.6-24.1) |  |
| 1 | 11.8(7.9-15.6) |  | 17.9(12.2-23.5) |  |
| 2 | 9.7(7.6-11.7) |  | 15.0(9.9-20.0) |  |
| Tumor location |  | 0.889 |  | 0.751 |
| Ut | 10.2(1.8-18.5) |  | 15.9(2.5-29.2) |  |
| Mt | 11.8(8.5-15.0) |  | 17.4(12.9-21.8) |  |
| Lt | 9.7(5.5-13.8) |  | 15.3(8.9-21.6) |  |
| Smoking history |  | 0.863 |  | 0.978 |
| Non-smoker | 9.9(6.1-13.6) |  | 15.0(8.3-21.6) |  |
| Smoker | 10.9(8.2-13.5) |  | 16.7(12.6-20.7) |  |
| Alcohol history | LR 0.652 | 0.722 |  | 0.813 |
| Never | 12.8(0.0-30.9) |  | 18.0(0.0-44.0) |  |
| Previous(≥6 mo.) | 11.8(7.9-15.6) |  | 18.3(13.1-23.4) |  |
| Current | 10.0(6.3-13.6) |  | 14.9(9.5-20.2) |  |
| Postoperative Stage |  | **0.001** |  | **0.001** |
| I | 24.1( 0.0–89.1 ) |  | 33.4( 0.0-111.2) |  |
| IIA +IIB | 13.1(7.9-18.2) |  | 20.3(15.9-24.6) |  |
| III | 8.6(5.1-12.0) |  | 11.9(3.8-19.9) |  |
| IV | 5.1(2.3-7.8) |  | 7.9(0.7-15.0) |  |

Abbreviations: PFS: progression-free survival; OS: overall survival; CI: confidence interval; ECOG: Eastern Cooperative Oncology Group; PS: performance status; Ut: upper thoracic esophagus; Mt: middle thoracic esophagus; Lt: lower thoracic esophagus.
