# Supplementary material for: DNA repair gene polymorphisms and clinical outcome of patients with primary small cell carcinoma of the esophagus
Source: Tumour Biol. 2014 Nov 6;36(3):1539–48. doi: 10.1007/s13277-014-2718-y (PMC4375303; doi:10.1007/s13277-014-2718-y)
Supplement: Supplementary file 2 — (DOCX 30 kb) [file 13277_2014_2718_MOESM2_ESM.docx]

**Supplemental Table S2-1 Association of *PARP1-Val762Ala* genotypes with patient characteristics**

|  | T/T[n(%)] | T/C+C/C[n(%)] | X^2^ | *P* |
| --- | --- | --- | --- | --- |
| Age (years) |  |  | 0.117 | 0.733 |
| < 60 | 23(53.5) | 27(50.0) |  |  |
| ≥ 60 | 20(46.5) | 27(50.0) |  |  |
| Gender |  |  | 1.083 | 0.298 |
| Male | 37(86.0) | 42(77.8) |  |  |
| Female | 6(14.0) | 12(22.2) |  |  |
| ECOG PS |  |  | 0.014 | 0.904 |
| 0 | 17(39.5) | 22(40.7) |  |  |
| 1+2 | 26(60.5) | 32(59.3) |  |  |
| Tumor location |  |  | 0.092 | 0.761 |
| Ut+ Mt | 30(69.8) | 32(59.2) |  |  |
| Lt | 13(30.2) | 22(40.8) |  |  |
| Smoking history |  |  | 0.947 | 0.330 |
| Non-smoker | 9(20.9) | 16(29.6) |  |  |
| Smoker | 34(79.1) | 38(70.4) |  |  |
| Alcohol history |  |  | 1.167 | 0.280 |
| Never+ Previous | 16(37.2) | 26(48.1) |  |  |
| Current | 27(62.8) | 28(51.9) |  |  |
| Postoperative Stage |  |  | 2.427 | 0.489 |
| I | 4(9.3) | 6(11.1) |  |  |
| II | 17(39.5) | 20(37.0) |  |  |
| III | 19(44.2) | 19(35.2) |  |  |
| IV | 3(7.0) | 9(16.7) |  |  |
