# Supplementary material for: DNA repair gene polymorphisms and clinical outcome of patients with primary small cell carcinoma of the esophagus
Source: Tumour Biol. 2014 Nov 6;36(3):1539–48. doi: 10.1007/s13277-014-2718-y (PMC4375303; doi:10.1007/s13277-014-2718-y)
Supplement: Supplementary file 3 — (DOCX 30 kb) [file 13277_2014_2718_MOESM3_ESM.docx]

**Supplemental Table S2-2 Association of *XRCC1-Arg194Trp* genotypes with patient characteristics**

|  | C/C[n(%)] | C/T+T/T[n(%)] | X^2^ | *P* |
| --- | --- | --- | --- | --- |
| Age (years) |  |  | 1.675 | 0.196 |
| < 60 | 31(57.4) | 19(44.2) |  |  |
| ≥ 60 | 23(42.6) | 24(55.8) |  |  |
| Gender |  |  | 0.265 | 0.607 |
| Male | 43(79.6) | 36(83.7) |  |  |
| Female | 11(20.4) | 7(16.3) |  |  |
| ECOG PS |  |  | 1.879 | 0.170 |
| 0 | 25(46.3) | 14(32.6) |  |  |
| 1+2 | 29(53.7) | 29(67.4) |  |  |
| Tumor location |  |  | 0.092 | 0.761 |
| Ut+ Mt | 40(74.1) | 22(51.2) |  |  |
| Lt | 14(25.9) | 21(48.8) |  |  |
| Smoking history |  |  | 2.075 | 0.150 |
| Non-smoker | 17(31.5) | 8(18.6) |  |  |
| Smoker | 37(68.5) | 35(81.4) |  |  |
| Alcohol history |  |  | 0.446 | 0.504 |
| Never+ Previous | 25(46.3) | 17(39.5) |  |  |
| Current | 29(53.7) | 26(60.5) |  |  |
| Postoperative Stage |  |  | 1.247 | 0.742 |
| I | 4(7.4) | 6(14.0) |  |  |
| II | 22(40.7) | 15(34.9) |  |  |
| III | 21(38.9) | 17(39.5) |  |  |
| IV | 7(13.0) | 5(11.6) |  |  |
