# Supplementary material for: DNA repair gene polymorphisms and clinical outcome of patients with primary small cell carcinoma of the esophagus
Source: Tumour Biol. 2014 Nov 6;36(3):1539–48. doi: 10.1007/s13277-014-2718-y (PMC4375303; doi:10.1007/s13277-014-2718-y)
Supplement: Supplementary file 4 — (DOCX 30 kb) [file 13277_2014_2718_MOESM4_ESM.docx]

**Supplemental Table S2-3 Association of *XRCC1-Arg399Gln* genotypes with patient characteristics**

|  | G/G[n(%)] | G/A+A/A[n(%)] | X^2^ | *P* |
| --- | --- | --- | --- | --- |
| Age (years) |  |  | 2.402 | 0.121 |
| < 60 | 23(44.2) | 27(60.0) |  |  |
| ≥ 60 | 29(55.8) | 18(40.0) |  |  |
| Gender |  |  | 0.034 | 0.854 |
| Male | 42(80.8) | 37(82.2) |  |  |
| Female | 10(19.2) | 8(17.8) |  |  |
| ECOG PS |  |  | 0.755 | 0.385 |
| 0 | 23(44.2) | 16(35.6) |  |  |
| 1+2 | 29(55.8) | 29(64.4) |  |  |
| Tumor location |  |  | 0.053 | 0.818 |
| Ut+ Mt | 32(61.5) | 30(66.7) |  |  |
| Lt | 20(38.5) | 15(33.3) |  |  |
| Smoking history |  |  | 0.553 | 0.457 |
| Non-smoker | 15(28.8) | 10(22.2) |  |  |
| Smoker | 37(71.2) | 35(77.8) |  |  |
| Alcohol history |  |  | 1.068 | 0.301 |
| Never+ Previous | 20(38.5) | 22(48.9) |  |  |
| Current | 32(61.5) | 23(51.1) |  |  |
| Postoperative Stage |  |  | 0.574 | 0.902 |
| I | 6(11.5) | 4(8.9) |  |  |
| II | 21(40.4) | 16(35.6) |  |  |
| III | 19(36.5) | 19(42.2) |  |  |
| IV | 6(11.5) | 6(13.3) |  |  |
