# Supplementary material for: DNA repair gene polymorphisms and clinical outcome of patients with primary small cell carcinoma of the esophagus
Source: Tumour Biol. 2014 Nov 6;36(3):1539–48. doi: 10.1007/s13277-014-2718-y (PMC4375303; doi:10.1007/s13277-014-2718-y)
Supplement: Supplementary file 5 — (DOCX 30 kb) [file 13277_2014_2718_MOESM5_ESM.docx]

**Supplemental Table S2-4 Association of *XPC-Lys939Gln* genotypes with patient characteristics**

|  | A/A[n(%)] | A/C+C/C[n(%)] | X^2^ | *P* |
| --- | --- | --- | --- | --- |
| Age (years) |  |  | 1.242 | 0.265 |
| < 60 | 28(57.1) | 22(45.8) |  |  |
| ≥ 60 | 21(42.9) | 26(54.2) |  |  |
| Gender |  |  | 1.195 | 0.274 |
| Male | 42(85.7) | 37(77.1) |  |  |
| Female | 7(14.3) | 11(22.9) |  |  |
| ECOG PS |  |  | 0.015 | 0.901 |
| 0 | 20(40.8) | 19(39.6) |  |  |
| 1+2 | 29(59.2) | 29(60.4) |  |  |
| Tumor location |  |  | 0.007 | 0.932 |
| Ut+ Mt | 28(57.1) | 34(70.8) |  |  |
| Lt | 21(42.9) | 14(39.2) |  |  |
| Smoking history |  |  | 1.490 | 0.222 |
| Non-smoker | 10(20.4) | 15(31.3) |  |  |
| Smoker | 39(79.6) | 33(68.7) |  |  |
| Alcohol history |  |  | 0.249 | 0.618 |
| Never+ Previous | 20(40.8) | 22(45.8) |  |  |
| Current | 29(59.2) | 26(54.2) |  |  |
| Postoperative Stage |  |  | 1.771 | 0.621 |
| I | 5(10.2) | 5(10.4) |  |  |
| II | 19(38.8) | 18(37.5) |  |  |
| III | 21(42.9) | 17(35.4) |  |  |
| IV | 4(8.2) | 8(16.7) |  |  |
