# Supplementary material for: DNA repair gene polymorphisms and clinical outcome of patients with primary small cell carcinoma of the esophagus
Source: Tumour Biol. 2014 Nov 6;36(3):1539–48. doi: 10.1007/s13277-014-2718-y (PMC4375303; doi:10.1007/s13277-014-2718-y)
Supplement: Supplementary file 6 — (DOCX 30 kb) [file 13277_2014_2718_MOESM6_ESM.docx]

**Supplemental Table S2-5 Association of *BRCA1-Lys1183Arg* genotypes with patient characteristics**

|  | G/G[n(%)] | G/A+A/A[n(%)] | X^2^ | *P* |
| --- | --- | --- | --- | --- |
| Age (years) |  |  | 0.029 | 0.864 |
| < 60 | 20(52.6) | 30(50.8) |  |  |
| ≥ 60 | 18(47.4) | 29(49.2) |  |  |
| Gender |  |  | 0.001 | 0.978 |
| Male | 31(81.6) | 48(81.4) |  |  |
| Female | 7(18.4) | 11(18.6) |  |  |
| ECOG PS |  |  | 0.294 | 0.588 |
| 0 | 14(36.8) | 25(42.4) |  |  |
| 1+2 | 24(63.2) | 34(57.6) |  |  |
| Tumor location |  |  | 0.969 | 0.325 |
| Ut+ Mt | 25(65.8) | 37(62.7) |  |  |
| Lt | 13(34.2) | 22(37.3) |  |  |
| Smoking history |  |  | 0.143 | 0.706 |
| Non-smoker | 9(23.7) | 16(27.1) |  |  |
| Smoker | 29(76.3) | 43(72.9) |  |  |
| Alcohol history |  |  | 3.495 | 0.062 |
| Never+ Previous | 12(31.6) | 30(50.8) |  |  |
| Current | 26(68.4) | 29(49.2) |  |  |
| Postoperative Stage |  |  | 3.133 | 0.372 |
| I | 5(13.2) | 5(8.5) |  |  |
| II | 12(31.6) | 25(42.4) |  |  |
| III | 14(36.8) | 24(40.6) |  |  |
| IV | 7(18.4) | 5(8.5) |  |  |
