# Supplementary material for: DNA repair gene polymorphisms and clinical outcome of patients with primary small cell carcinoma of the esophagus
Source: Tumour Biol. 2014 Nov 6;36(3):1539–48. doi: 10.1007/s13277-014-2718-y (PMC4375303; doi:10.1007/s13277-014-2718-y)
Supplement: Supplementary file 7 — (DOCX 30 kb) [file 13277_2014_2718_MOESM7_ESM.docx]

**Supplemental Table S2-6 Association of *BRCA2-Asn372His* genotypes with patient characteristics**

|  | T/T[n(%)] | T/G+G/G[n(%)] | X^2^ | *P* |
| --- | --- | --- | --- | --- |
| Age (years) |  |  | 1.749 | 0.186 |
| < 60 | 39(55.7) | 11(40.7) |  |  |
| ≥ 60 | 31(44.3) | 16(59.3) |  |  |
| Gender |  |  | 1.846 | 0.174 |
| Male | 55(78.6) | 24(88.9) |  |  |
| Female | 15(21.4) | 3(11.1) |  |  |
| ECOG PS |  |  | 0.735 | 0.391 |
| 0 | 30(42.9) | 9(33.3) |  |  |
| 1+2 | 40(57.1) | 18(66.7) |  |  |
| Tumor location |  |  | 0.737 | 0.390 |
| Ut+ Mt | 45(64.3) | 17(63.0) |  |  |
| Lt | 25(35.7) | 10(37.0) |  |  |
| Smoking history |  |  | 1.029 | 0.310 |
| Non-smoker | 20(28.6) | 5(18.5) |  |  |
| Smoker | 50(71.4) | 22(81.5) |  |  |
| Alcohol history |  |  | 0.100 | 0.752 |
| Never+ Previous | 31(44.3) | 11(40.7) |  |  |
| Current | 39(55.7) | 16(59.3) |  |  |
| Postoperative Stage |  |  | 0.260 | 0.967 |
| I | 7(10.0) | 3(11.1) |  |  |
| II | 27(38.6) | 10(37.0) |  |  |
| III | 28(40.0) | 10(37.0) |  |  |
| IV | 8(11.4) | 4(14.8) |  |  |
